# The Impact of Sex and Age on the Prevalence of Clinically Relevant Sensitization and Asymptomatic Sensitization in the General Population

**DOI:** 10.1007/s00005-016-0425-7

**Published:** 2016-09-20

**Authors:** Anna Dor-Wojnarowska, Jerzy Liebhart, Jadwiga Miecielica, Marek Rabski, Andrzej Fal, Bolesław Samoliński, Marita Nittner-Marszalska

**Affiliations:** 1 Department of Internal Diseases and Allergology, Wroclaw Medical University, M. Skłodowskiej-Curie 66, 50-369 Wrocław, Poland; 2 Department of Prevention of Environmental Hazards and Allergology, Medical University, Warsaw, Poland

**Keywords:** Asymptomatic sensitization, Population study, “Prick” skin test, Total IgE

## Abstract

The objective of our study was to evaluate the impact of sex and age on the prevalence of sensitization to inhalant allergens. The study was performed as a part of Polish Epidemiology of Allergic Diseases study, and data concerning citizens of Wroclaw were analyzed. The participants were divided into three age groups (6–7, 13–14, and 20–44 years) with a subdivision according to sex. We randomly selected 1409 individuals, 439 people complied; the complete set of tests was performed on 421 of them. We found that 37.7 % of the study population demonstrated sensitization to at least one of the allergens tested. Positive skin tests were found more frequently in males than in females (*p* = 0.003); among 6–7-year-old children, the sensitization was independent of sex (*p* = 0.26), while in two other groups, it was higher in males (*p* = 0.002 and *p* = 0.03, respectively). Clinically asymptomatic sensitization (AS) was found more often in females than in males (*p* = 0.04). The higher rate of AS in women was observed only in the two younger age groups, while in the 20–44-year-old group AS did not differ between the sexes (*p* = 0.72). Female sex hormones may contribute to a later change in the nature of sensitization from clinically asymptomatic to symptomatic. Further studies are needed to confirm the results of our study.

## Introduction

The term sensitization indicates the production of specific IgE (sIgE) antibodies directed against exogenous allergenic molecules. The initiation of sIgE production, resulting from the interaction between genetic predisposition, exposure to environmental factors, and properties of the allergen are a major contributing factor to the development of allergy signs and symptoms. The sensitization process itself is a stage, which can, but does not always lead to the development of the symptoms of allergic rhinitis, asthma, and a number of other allergic diseases. It is currently unknown why some people who are sensitized to a given allergen develop allergic symptoms, while others tolerate it without symptoms. However, it appears to be the result of an interaction between the genetic background and the epigenetic make-up of the host (van Ree et al. 2014).

Numerous epidemiological studies have shown an increasing incidence of allergies worldwide. It is estimated that sensitization to inhalant allergens occurs among 17–55 % of people (Bousquet et al. 2007). It is important to identify the phenotype of patients in whom this process is more common and clinically significant. Several factors are believed to be responsible for the different rates of sensitization and clinical reactivity to allergens or its absence in sensitized people, including individual factors (age, sex, and family history of atopy) as well as immunological factors (serum levels of total IgE, sIgE, or IgG, the epitope specificity of IgE, mono- vs poly-sensitization, and the Treg/Th1/Th2 balance). None of these factors seem to have a decisive influence by themselves on the occurrence of clinically manifested symptoms of sensitization (Migueres et al. 2014). In this context, a group of studies evaluating the relationship between the frequency of sensitization/allergy symptoms and sex has been conducted (Wüthrich et al. 2013; Govaere et al. 2007).

The theoretical rationale for the impact of sex on the sensitization process and the occurrence of allergies is the influence of estrogens on the humoral response, on the immediate-type hypersensitivity reactions, and on the delayed-type reactions (Chen et al. 2008; Jensen-Jarolim and Untersmayr 2008). Recent experimental data indicate that estrogens potentiate the effector phase of IgE-mediated allergic reactions by augmenting the activation of mast cells and, consequently, increasing the secretion of inflammatory mediators by mast cells and other effector cells (Cocchiara et al. 1990, 1992; Eisenberg et al. 2008; Lang 2004). Alternately, this effect may be due to the presence of estrogen receptors, such as estrogen receptor α, on most cells involved in the immune response, including the immediate response. Clinical data supporting the role of sex in the sensitization process include higher incidences of urticaria, anaphylaxis, food allergy, and asthma in women than in men (Lenoir 1987; Poulos et al. 2007; Schäfer et al. 2001).

The main objective of our study was to evaluate the impact of sex and age on the prevalence of sensitization to inhalant allergens in the population of the Lower Silesia, which is a region in south-western Poland.

## Materials and Methods

The study was performed as a part of the Epidemiology of Allergic Diseases in Poland (ECAP) study, which is a continuation of two international studies: the European Community Respiratory Health Survey II (ECRHS II) and the International Study of Asthma and Allergies in Childhood (ISAAC). For the purpose of this study, the ECAP data concerning citizens of Wroclaw were analyzed. The study protocol was approved by the Bioethics Committee of Warsaw Medical University as well as by the Inspector General for the Protection of Personal. The detailed methodology of the ECAP study has been previously described (Ponińska et al. 2011; Sybilski et al. 2015). Briefly, the ECAP study used the same methodology as the ISAAC (includes populations aged 6–7 and 13–14 years) and ECRHS II (includes a population aged 20–44 years) studies. ECAP included randomly selected populations from all the three of these age groups (ages 6–7, 13–14, and 20–44 years). The study was performed in major metropolitan areas of Poland (Katowice, Wrocław, Lublin, Gdańsk, Warszawa, Poznań, and Białystok). In the ECAP study, all the respondents were randomly chosen from the National Polish Social Security Number database. Then, within each of these groups, simple sampling was performed. The number of randomly selected individuals from each stratum was proportional to the part of the stratum in the studied population.

In this study, we analyzed the results from the part of the ECAP study that was conducted in Wroclaw. During the study period between October 2007 and April 2008, the city had 636,854 residents, including 298,556 men. The participants were split into three age groups (6–7, 13–14, and 20–44 years) with a subdivision according to sex, for a total of six groups. We randomly selected 1409 individuals and requested that they come to the outpatient clinic to be given more detailed examinations. From this group, 439 people complied, and the complete set of planned examinations and tests was performed on 421 of them.

During the outpatient visit, several examinations and tests were performed. Patients received a medical examination, which included a medical history taken with the help of a questionnaire, a physical examination, and verification of their diagnosis. In addition, skin prick tests were performed with 15 aeroallergens, and a blood sample was taken (20 mL in adults and 12 mL in children) to determine each subject’s IgE antibody levels. The participants or their legal representatives signed informed consent forms before the abovementioned exams were performed, one for the physical examination and a separate consent form for the blood test.

### Study Questionnaire

In the present study, the ECRHS II questionnaire and ISAAC study questionnaire were applied jointly, which enabled us to compare the results between the adult group and the child group. The total number of questions between these questionnaires was about 400; however, owing to the use of an advanced filter system, the respondents answered only a portion of these questions. The questions analyzed in this study were related to environmental factors, the participant’s specific allergic disease symptoms, the presentation of symptoms during contact with inhalant allergens, and the participant’s present medical diagnosis.

The questionnaire underwent a double validation. The first validation was conducted in 2003, in Świdnica, Lower Silesia, in a 1000 person group of respondents. The second validation was carried out in May 2006 in a group of 150 citizens of Warszawa (Majkowska-Wojciechowska et al. 2007; Samoliński 2007).

### Skin Prick Tests

Skin prick tests were performed with the following allergens: hazel, alder, birch, grasses/grains, rye, tarragon, plantain, *Alternaria*, *Cladosporium*, group I molds (*Alternaria tenuis*, *Botrytis cinerea*, *Cladosporium herbarum*, *Culvularia lunata*, *Fusarium moniliforme*, and *Helminthosporium*), group II molds (*Aspergillus fumigatus*, *Mucedo mucor*, *Penicillium notatum*, *Rhizopus nigricans*, *Serpula lacrymans*, and *Pullularia pullulans*), *Dermatophagoides pteronyssinus*, *Dermatophagoides farinae*, dog, and cat (Allergopharma—Nexter, Germany). All tests were performed by the same person, who had many years of experience in this field. Tests were performed on both forearms. Lancets (Allergopharma) were used to puncture the skin, and the skin prick tests were performed according to The European Academy of Allergy and Clinical Immunology recommendations (Dreborg and Frew 1993). These tests were routinely conducted with both a positive and a negative control. Wheal reactions with a mean diameter of 3 mm or more were regarded as positive, if the control solutions showed the expected results (wheal size of at least 3 mm for the positive control and of less than 3 mm for the negative control). Patients were excluded if their responses to control solutions were not adequate. The result was read after 15 min and was calculated as the sum of the longest diameter of the bubble and the diameter perpendicular to it, divided by two. To determine the type of sensitization, the allergens were grouped into four categories: pollens (hazel, alder, birch, grasses/grains, rye, *Artemisia*, and plantain), mites (*D. pteronyssinus* and *D. farinae*), molds (groups I and II), and animal allergens (dog and cat).

### Total IgE

The concentration of total IgE in the serum was determined using reagents from the Phadia CAP System according to the manufacturer’s instructions. The obtained data are presented in international units (IU)/mL.

### Asymptomatic Sensitization

Asymptomatic sensitization was defined as the presence of sIgE antibodies detectable in skin tests or serological tests in patients showing no clinical allergic symptoms to a specific allergen.

### Allergic Diseases Diagnoses

The clinical diagnoses of asthma (atopic or nonatopic), intermittent allergic rhinitis (i.e., with symptoms present fewer than 4 days per week or for fewer than four consecutive weeks), persistent allergic rhinitis (i.e., with symptoms present more than 4 days per week and for more than four consecutive weeks), and atopic dermatitis were based on the International Global Initiative for Asthma (GINA Report 2012) guidelines, Allergic Rhinitis and its Impact on Asthma criteria (Bousquet et al. 2001), and the criteria of Hanifin and Rajka (1980), respectively. In addition, a history of food allergy, drug allergy, insect bite allergy, urticaria, Quincke’s edema, and/or other chronic diseases was obtained.

### Statistical Analysis

The results were analyzed using the Statistica 9 software. The Shapiro–Wilk test was used to examine the data for distribution normality. Owing to the lack of a normal distribution of studied parameters, non-parametric tests were used. To describe the data, the median (Me) and the lowest and highest values (min–max) are shown. Comparisons of two independent samples were performed using Kolmogorov–Smirnov *U* tests or Chi-squared tests with Yates’ correction. For the comparisons of three groups of patients, we used the analysis of variance tests, Kruskal–Wallis rank tests, or Chi-squared tests. For all the tests, *p* values of <0.05 were considered statistically significant. Receiver operator characteristic tests were applied for the evaluations of the usefulness of the analyzed parameters for the discrimination between allergic and non-allergic groups and between symptomatic and non-symptomatic allergic patients.

## Results

### Demographic Data

The demographic data for the 421 study participants (224 women) are presented in Table 1.

**Table 1:** Demographic and clinical data

Parameter	6–7 years	13–14 years	20–44 years	*p*
*N*	130	132	159	
Sex (female/male)	(52/78)	(69/63)	(103/56)	
Age years Me (min–max)	7 (6–7)	13 (13–14)	29 (20–44)	
BMI Me (min–max)	15 (10–21)	19 (13–27)	23 (16–35)	0.0002**
Asthma atopic % (*N*)	1.5 % (2)	4.5 % (6)	3.1 % (5)	0.37*
Asthma nonatopic % (*N*)	4.6 % (6)	1.5 % 9 (2)	0.0 % (0)	0.15*
Allergic rhinitis intermittent % (*N*)	6.9 % (9)	7.6 % (10)	10.1 % (16)	0.58*
Allergic rhinitis persistent % (*N*)	11.5 % (15)	16.7 % (22)	15.1 % (24)	0.48*
Healthy^a^ % (*N*)	66.9 % (87)	68.9 % (91)	66.0 % (105)	0.86*
Sensitization % of examined (*N*)	28.4 % (37)	39.4 % (52)	44.0 % (70)	0.02*
Asymptomatic sensitization^b^ % of sensitized (*N*)	35.1 % (13)	36.5 % (19)	42.0 % (29)	0.73*
Total IgE (IU/mL) Me (min–max)	22.9 (0.8–808.3)	13.8 (1.5–679.1)	9.9 (0.2–1824.1)	0.0002**

*N* number of examined patients, *Me* median, *Min* minimum, *Max* maximum, *%* percentage of patients, *p p* value

* Chi-square test; ** Kruskal–Wallis test

^a^Without any allergic disease and symptoms

^b^Asymptomatic sensitization was defined as the presence of sIgE antibodies detectable in skin tests or serological tests in patients showing no clinical allergic symptoms to a specific allergen

### Age and Sex Differences in the Prevalence of Positive Skin Tests (Sensitization)

**Fig. 1:**
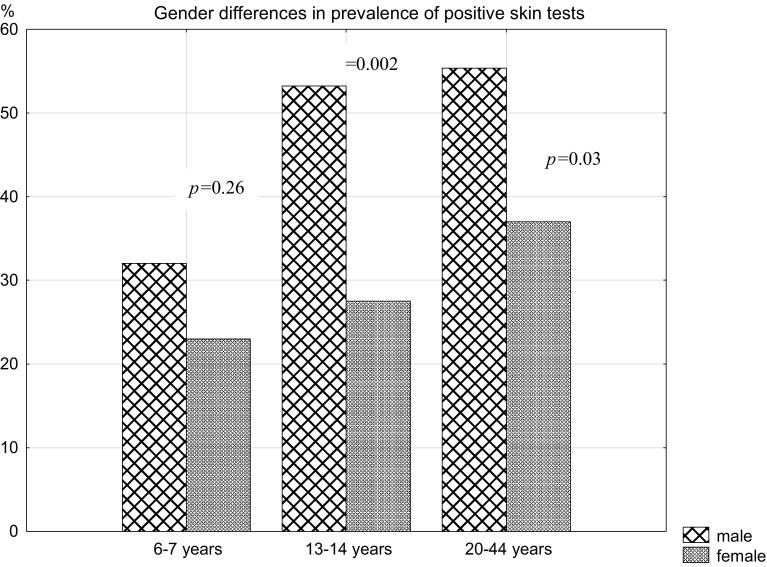
Gender differences in the prevalence of positive skin tests. Wheal reactions with a mean diameter of 3 mm or more were regarded as positive if the control solutions showed the expected results (wheal size of at least 3 mm for the positive control and of less than 3 mm for the negative control)

We found that 37.7 % of the study population demonstrated sensitization (allergy) to at least one of the allergens tested. Overall, positive skin tests were found more frequently in male (45.2 %) than in female individuals (31.2 %; *p* = 0.003). Among 6–-year-old children, the prevalence of sensitization was independent of sex (*p* = 0.26). However, in the 13–14- and 20–44-year-old age groups, the prevalence of sensitization was higher in male individuals (*p* = 0.002 and *p* = 0.03, respectively; Fig. 1).

The percentage of participants with positive skin tests increased with age in the analyzed groups, from 28.4 % in the group of 6–7-year-old children to 39.4 % in 13–14-year-old individuals to 44.0 % in the population of 20–44-year-old adults (*p* = 0.02). This increase in the frequency of sensitization with increasing age was demonstrated only for male patients (*p* = 0.01); the prevalence of allergies in the 6–7-, 13–14-, and 20–44-year-old age groups in men was as follows: 32, 53, and 55 %, respectively. In the female population, while there was a trend toward a similar increase (23, 27, and 38 %, respectively), these differences did not reach statistical significance (*p* = 0.12).

### Age and Sex Differences in the Prevalence of Mono- and Poly-Sensitization

When all groups were combined, there was no difference (*p* = 0.18) in the prevalence of monovalent and polyvalent sensitization between female (78.6 %) and male (86.5 %) individuals. However, in the 6–7-year-old group, boys had polyvalent allergies more often than girls (88 % of boys vs 58 % of girls, *p* = 0.04). In older age groups, the prevalence of polyvalent sensitization did not differ between the two sexes, either among 13–14 years (*p* = 0.46) or 20–44 years (*p* = 0.7). In male patients, the rates of allergy to pollens (32 vs 21 %, *p* = 0.01), mites (29 vs 20 %, *p* = 0.03), and molds (9 vs 3 %, *p* = 0.01) were higher than those in female patients.

### Sex Differences in the Prevalence of Asymptomatic Sensitization

**Fig. 2:**
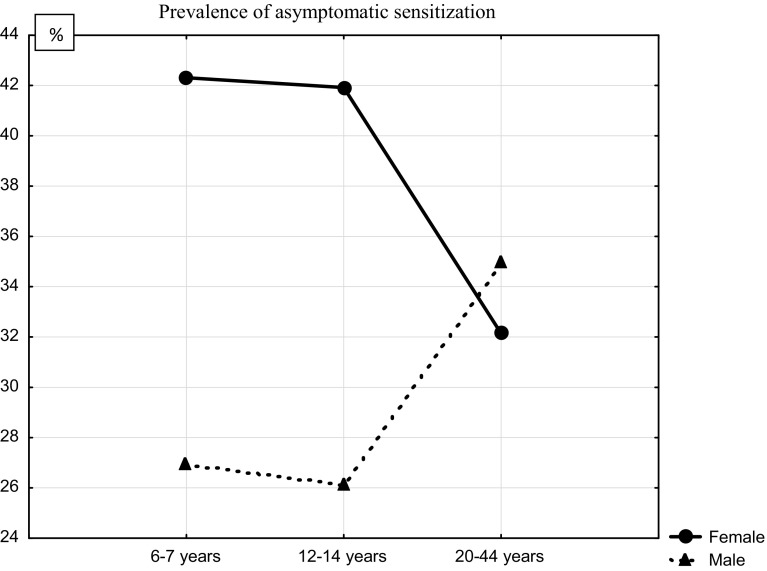
Gender differences in the prevalence of asymptomatic sensitization. Asymptomatic sensitization was defined as the presence of sIgE antibodies detectable in skin tests or serological tests in patients showing no clinical allergic symptoms to a specific allergen

Clinically asymptomatic sensitization was found among 38.4 % of participants who were sensitized to at least one allergen, and this occurred more often in female (41.4 %) than in male (35.9 %) individuals (*p* = 0.04). The higher rate of asymptomatic sensitization in women than in men was observed only in the two younger age groups (42.3 and 41.9 % in 6–7- and 13–14-year-old girls, respectively, vs 26.9 and 26.1 % in 6–7- and 13–14-year-old boys, respectively), while in the 20–44-year-old group, the prevalence of asymptomatic sensitization was not different between the two sexes (32.1 % of male vs 34.9 % of female individuals, *p* = 0.72; Fig. 2).

### Sex Differences in Total IgE Concentration

In the whole group of participants, the concentration of total IgE was higher (*p* = 0.023) in male individuals (Me: 16; 95 % confidence interval 0.78–1824.15 IU/mL) than in female individuals (Me: 11; 95 % confidence interval 0.24–679.19 IU/mL).

**Fig. 3:**
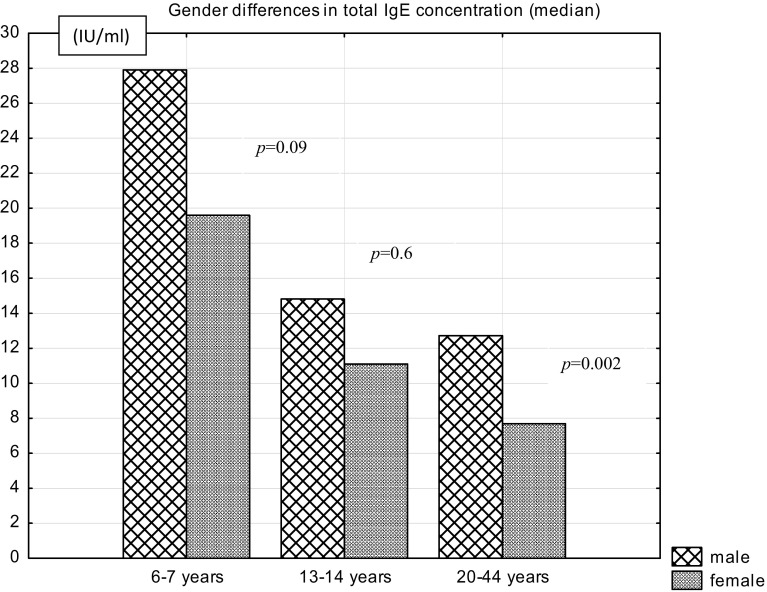
Gender differences in total IgE concentration

The analysis of the total IgE level depending on sex in different age groups showed no significant difference in the size of this parameter in younger age groups (6–7 and 13–14 years) but a higher total IgE level in men than in women in the group of 20–44 years (Fig. 3).

**Fig. 4:**
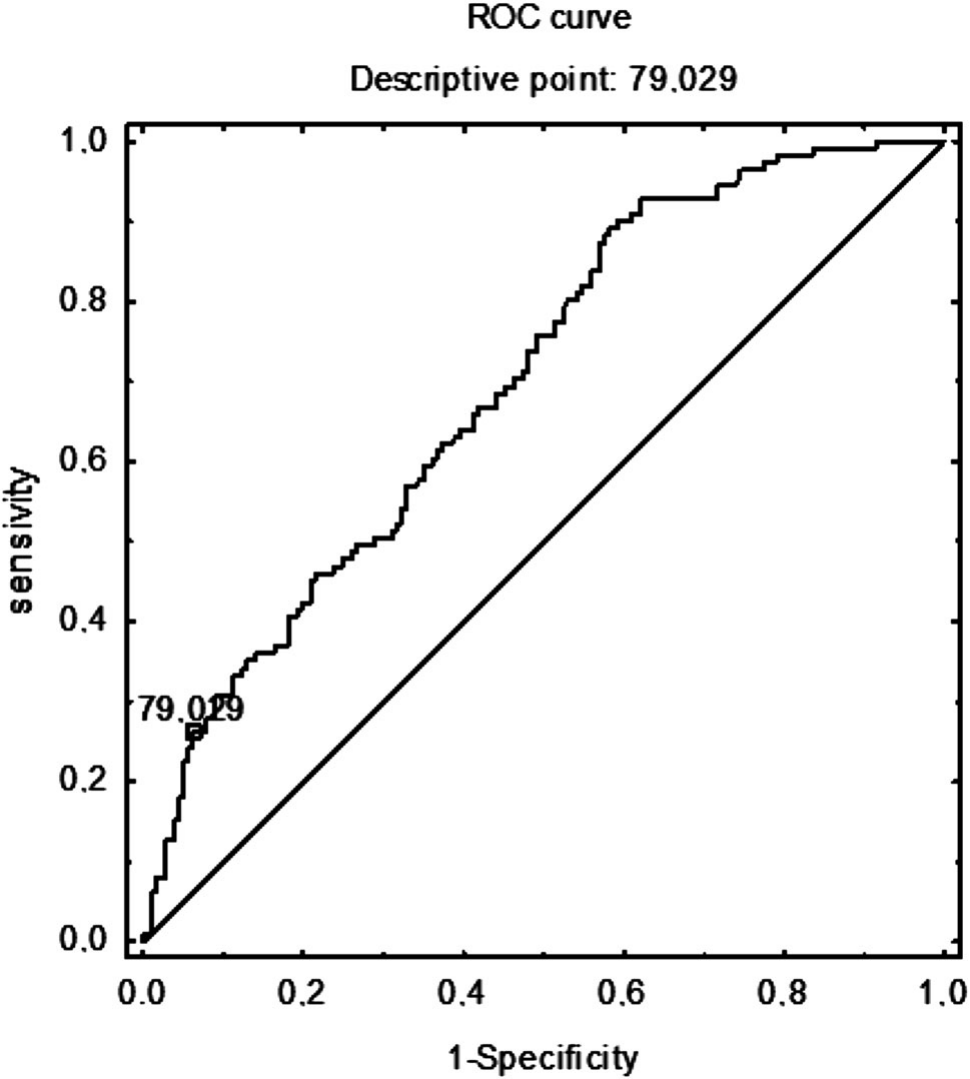
Receiver operator characteristics (ROC) curve of total IgE (IU/mL): atopy vs nonatopy. On the basis of the receiver operator characteristics curve, a total IgE threshold of 79 IU/mL was adopted as the best differentiating value between people who are sensitized and non-sensitized, with 93 % specificity and 26 % sensitivity (area under the curve: 0.69)

On the basis of the receiver operator characteristics curve, a total IgE threshold of 79 IU/mL was adopted as the best differentiating value between people who are sensitized and non-sensitized, with 93 % specificity and 26 % sensitivity (area under the curve: 0.69; Fig. 4).

### Differences in the Total IgE Level between Allergic and Asymptomatic Subjects

In the asymptomatic sensitization group, the total IgE level was lower (Me, min–max: 11.9, 3.14–1824.1) than that in the symptomatic group (Me, min–max: 35.0, 2.0–512; *p* = 0.008). This correlation occurred in both male (*p* = 0.007) and female individuals (*p* = 0.05). Symptoms were more frequent in subjects with polyvalent sensitization (*p* = 0.0003), particularly in patients allergic to mites (*p* = 0.007) and animal dander (*p* = 0.04).

**Fig. 5:**
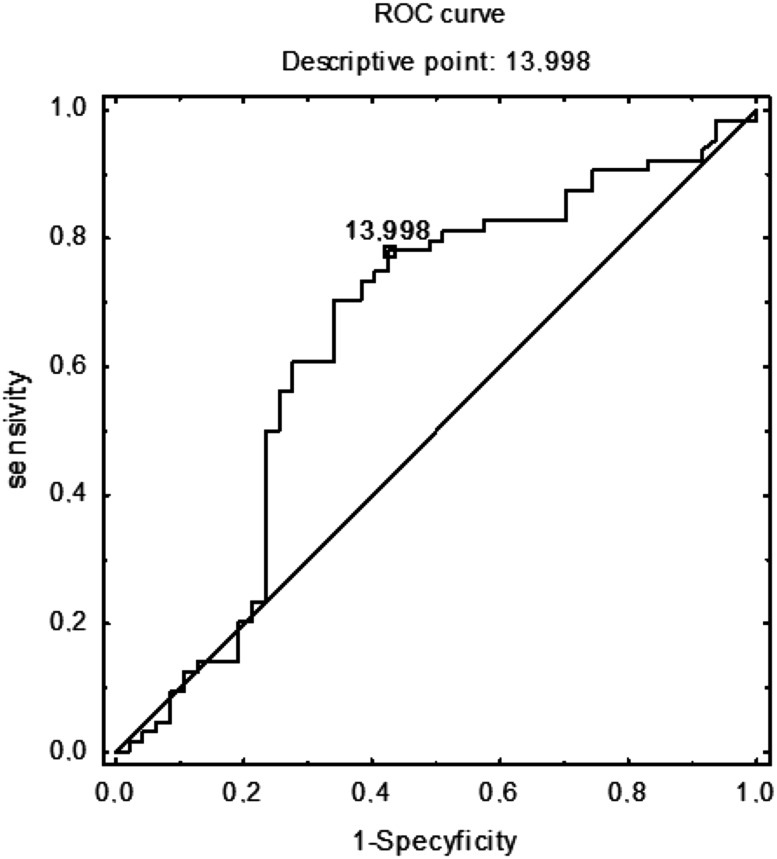
ROC (receiver operator characteristics) curve of total IgE (IU/mL): atopic and asymptomatic individuals vs atopic and symptomatic. The cut-off point of 14 IU/mL classified atopic individuals as asymptomatic or symptomatic with 57 % specificity and 78 % sensitivity (area under the curve: 0.65)

Next, we attempted to determine the level of total IgE that would indicate the possible existence of asymptomatic sensitization with the maximum sensitivity and specificity. The cut-off point of 14 IU/mL classified atopic individuals as asymptomatic or symptomatic with 57 % specificity and 78 % sensitivity (area under the curve: 0.65; Fig. 5).

## Discussion

The aim of our study was to evaluate the effect of sex on the prevalence of sensitization to inhalant allergens. We focused on the phenomenon of clinically asymptomatic sensitization and determining the factors that may influence it. While the prevalence of allergies, their association with geographical regions, and their relationship to age and sex have already received a great deal of attention, the question of the influence of age and sex on the “symptomatic” or “asymptomatic” character of the sensitization has not been widely explored.

Here, we demonstrate that, overall, women show a lower prevalence of sensitization (either symptomatic or asymptomatic) than men. This difference is especially marked in the younger age groups as 13–14-year-old girls presented clinically asymptomatic sensitization more frequently than similarly aged boys. However, this sex difference was only temporary, because we found no difference in the prevalence of asymptomatic sensitization between women and men in the 20–44-year-old age group. Moreover, in the female study groups, the occurrence of asymptomatic sensitization was the lowest in the oldest age group (20–44 years), while the opposite trend was observed in male individuals; the asymptomatic sensitization percentage in the oldest age group of men was higher than those in the other two male groups.

Our results may indicate a significant effect of estrogen on the process of clinical manifestations of allergy. The increase in the percentage of patients with symptomatic allergies among women aged 20–44 years compared with that in younger groups of the same sex may suggest that the high levels of estrogen present in the mature age group (20–44 years) may be a factor influencing the clinical expression and the clinically manifested symptoms of sensitization. The effect of estrogen may be biphasic. Low levels of estrogen could be a limiting factor in the clinical expression of allergy in sensitized female individuals, while its high levels could predispose individuals to the clinically manifested symptoms of sensitization. Similar conclusions were drawn by Liebhart et al. (2014) from the results of a big epidemiological study. Although both studies have different objectives, their conclusions are concordant. Liebhart et al. (2014) reported differences in the prevalence of allergic diseases (asthma, allergic rhinitis, atopic and contact dermatitis, and drug allergy) at young ages (higher prevalence in boys than girls) and adulthood (higher prevalence in women than men). On the basis of their results, the investigators suggested an influence of sex hormones in the clinical manifestation of their participants’ allergies. In theory, androgens can also influence the immune system differently from female sex hormones; however, data on this topic are currently scarce.

Other results from our study also correspond with the results of previous larger epidemiological studies, which we believe confirms the validity of our remaining observations. In the initial study, ECRHS among 11,355 participants, sensitization was observed in 32.2–43 %, depending on the country and geographic variations (Pearce et al. 2000), and we similarly observed sensitization in 37.7 % of our respondents. In addition, a similar result was obtained in a US study in which a positive skin test to at least one allergen was demonstrated in 54.3 % of the population (10,863 respondents aged between 6 and 59 years) (Arbes et al. 2005). Furthermore, Govaere et al. (2009) reported a similar percentage of sensitization in groups of children of comparable ages. Unfortunately, the authors of that study did not present data on the prevalence of asymptomatic sensitization in their study group, nor did they present data on the prevalence of sensitization in age subgroups or provide data on the prevalence of sensitization separately for boys and girls. The lack of this data prevents us from directly comparing our results with theirs (Govaere et al. 2009).

As in the previous studies, we found an increasing proportion of people with allergies in sequential age categories (Salo et al. 2014; Wüthrich et al. 2013). In our study, the increased percentage of sensitized participants in higher age groups was observed only in male individuals, not in female individuals. In addition, in our study, as in others, a higher prevalence of polyvalent rather than monovalent sensitization was observed as was a greater percentage of sensitization in male teenagers and men than in the corresponding age groups of girls and women (Hoppin et al. 2011). Allergies to *D. pterynossinus* and grass pollen were the most frequently observed allergies in our study. Similar findings were shown in the ECHRS I study (21.7 %) (Bousquet et al. 2007).

Our results indicate that the sensitization was asymptomatic in 38.4 % of the patients who were sensitized to at least one allergen. This result is similar to the results of the GA2LEN skin test study, which was carried out using a very similar methodology (Burbach et al. 2009; Heinzerling et al. 2009). In a study performed on the Danish population, 43 % of the individuals sensitized to inhalant allergens presented no respiratory symptoms (Kerkhof et al. 2000). A similar proportion of patients with asymptomatic allergies was observed in a study by Hoppin et al. (2011), in which 37 % of the 8334 participants presented with asymptomatic sensitization. A larger proportion of such patients was found in the study by Burbach et al. (2009). In this multicenter study conducted within the GA2LEN skin test study I, the authors showed that up to 50–95 % of the 3034 participants (depending on the type of allergen and country) presented clinically insignificant sensitization (Heinzerling et al. 2009).

Our results show that symptomatic allergies were presented more often in patients with polyvalent sensitization than in those with monovalent sensitization. This observation confirms reports by other authors. In their analysis of the factors that contribute to the occurrence of asymptomatic allergy, Bousquet et al. (2007) stated that people with asymptomatic sensitization were younger than those with a clinically relevant allergy, that their allergy was more often monovalent, and that they were less likely to have a positive family history of atopy. This idea was also confirmed by the results of Hoppin et al. (2011), who demonstrated that asymptomatic sensitization appeared more often in younger age groups.

Our study has some limitations. The first is its cross-sectional nature, which means that, unlike cohort studies, our evaluation of sensitization prevalence was carried out at a single time point; therefore, it has some limitations in estimating trends and in its assessment of risk factors. The second limitation concerns the small number of investigated individuals in the study groups; larger numbers of studied individuals would improve the reliability of our results. However, despite these drawbacks, the results of our study seem to be viable, as they strongly support findings from the previous research. In addition, a major strength of our investigation is the original finding indicating the existence of a sex advantage of young female individuals over young male individuals with respect to the prevalence of asymptomatic sensitization to inhalant allergens.

In conclusion, we believe that female sex hormones active during adolescence may contribute to a later change in the nature of sensitization, ranging from clinically asymptomatic to symptomatic. Further studies are needed to confirm the results of our study.
